# The Role of Gram Staining in Staphylococcal Scalded Skin Syndrome

**DOI:** 10.7759/cureus.7624

**Published:** 2020-04-10

**Authors:** Dan Morgenstern-Kaplan, Rodrigo Fonseca-Portilla, Enrique Konstat-Korzenny, Ariel Cohen-Welch

**Affiliations:** 1 Medicine, Universidad Anáhuac Mexico, Mexico City, MEX

**Keywords:** scalded skin syndrome, staphylococcus aureus, ssss, desquamation, dermatology, gram stain

## Abstract

Staphylococcal scalded skin syndrome (SSSS) is a severe blistering disease common in children. The diagnosis of SSSS is often difficult to distinguish from other blistering diseases in children. Here, we report a case of SSSS with a particular diagnostic step to elucidate the disease, which is the Gram stain. We propose the use of the Gram stain as a cost-effective diagnostic step in SSSS to shorten the time from presentation to treatment, especially in resource-limited areas.

## Introduction

Staphylococcal scalded skin syndrome (SSSS) is a toxin-mediated epidermolytic disease that occurs mainly in infants and children. Certain strains of* Staphylococcus aureus* (*S. aureus*) release epidermolytic toxins A and B (ET-A and ET-B), which bind to desmoglein-1, causing blistering, acantholysis and desquamation of the skin. The disease causes exquisite pain and usually requires hospitalization, due to age at presentation and possible complications (sepsis, dehydration and electrolyte abnormalities) [1].

## Case presentation

**Figure 1:**
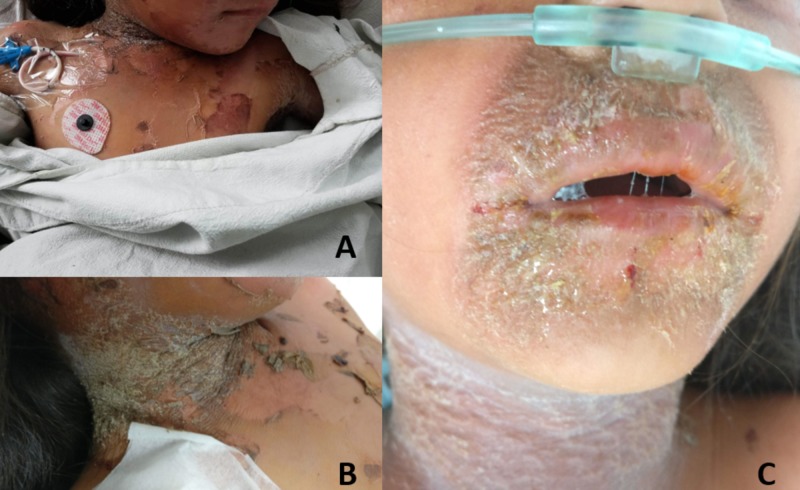
Photo montage – clinical pictures 1A. Patient with characteristic erythematous blistering skin lesions and acantholysis in the frontal chest area. 1B. Crusted lesion in the lateral cervical area with signs of previous blistering and erosion. 1C. Thick crusting and radial fissuring in the perioral area; mucous membranes were not involved.

A previously healthy, immunocompetent four-year-old girl presented to the emergency department with fever, malaise and several blistering lesions that appeared after two days of pain and erythema in the neck and extremities. The lesions started as flaccid bullae that desquamated and afterwards crusted. They were localized in the neck, extremities and perioral and periorbital areas, with copious purulent secretions around the nares and eyes. All the lesions were painful and desquamated when pressure was applied (positive Nikolsky sign) (Figure 1). 

**Figure 2:**
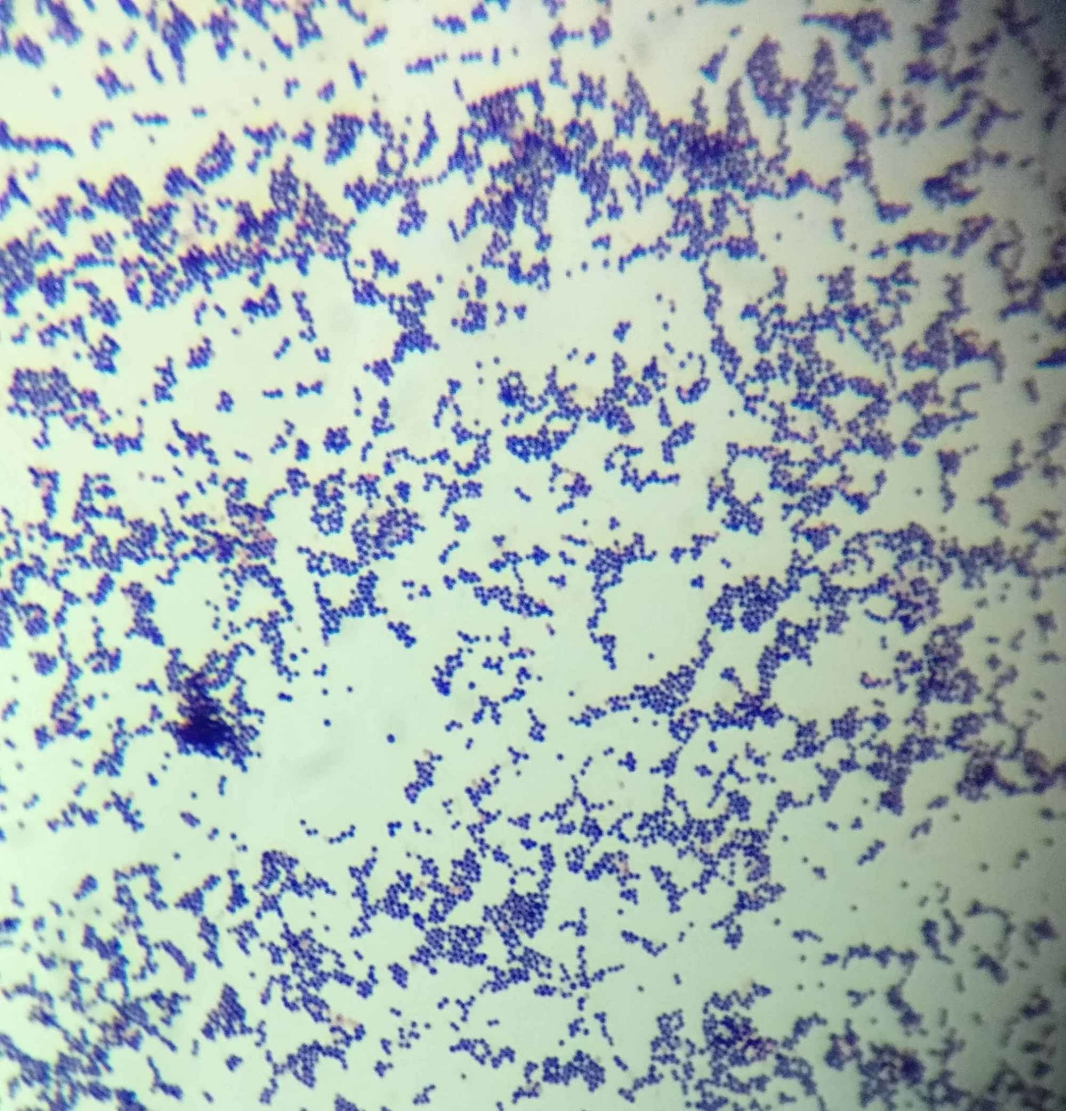
Gram-positive cocci in clusters on direct Gram stain

The patient's guardian denied any previous use of medications; nonetheless, the differential diagnosis of Stevens-Johnson syndrome/toxic epidermal necrolysis (SJS/TEN) and SSSS was considered as it was difficult to clinically distinguish them from the patient's presentation. IV lines were placed for hydration and pain management with acetaminophen. A sample of the ophthalmic and perioral secretions was obtained for Gram staining and culture. The Gram stain showed gram-positive cocci in clusters, supporting the diagnosis of SSSS (Figure 2). Antibiotic treatment with IV vancomycin was begun empirically. The patient improved significantly in the following 3-4 days. The culture yielded methicillin-sensitive *S. aureus* (MSSA); therefore, the ensuing antibiotic treatment was switched to oral dicloxacillin, and topical fusidic acid was prescribed. After 10 days of treatment, no new cutaneous lesions appeared; most of the lesions were healed and the pain subsided. The patient was discharged home.

## Discussion

SSSS is a relatively common disease in infants, and while manageable with antibiotics and analgesia, the diagnosis can be difficult as other dermatological processes may present a similar clinical picture, such as burns, bullous impetigo and the spectrum of SJS/TEN. Although diagnosis of SSSS is mainly clinical, laboratory tests may be performed to confirm it [1]. Here we propose the use of Gram staining as a fast and cost-effective diagnostic tool to differentiate the previously mentioned differential diagnoses, especially in resource-limited areas.

If Gram staining is performed, the usual findings compatible with* S. aureus* would yield Gram-positive cocci in clusters [2]. With a high degree of suspicion, finding the bacteria would provide additional evidence to start empiric therapy. It is important to note that the source of the sample must not be from bullae or exfoliative lesions, because no bacteria will be found as it is a toxin-mediated disease. Samples must be taken from conjunctiva, nasopharynx, umbilical stump, ear canal or the diaper area in the case of infants [1]. In most cases, the culture and antibiogram yield the preferred antibiotic to use; however, empiric therapy against MSSA should not be delayed, due to the low prevalence of methicillin-resistant *S. aureus*(MRSA) in SSSS [3]. Hence, the use of Gram staining is a quick and effective diagnostic step that could shorten the time between presentation and treatment.

## Conclusions

Here, we presented a patient with SSSS, diagnosed and distinguished from other differentials with a Gram stain, which is a cheap, fast, convenient but not a widely used method for diagnosis. This tool can help reduce the time between presentation, diagnosis and treatment of this disease; hence, we recommend the use of the Gram stain to clinicians and emergency personnel everywhere, especially in areas without specialized facilities for more sophisticated diagnostic methods.
